# Dynamic oceanography determines fine scale foraging behavior of Masked Boobies in the Gulf of Mexico

**DOI:** 10.1371/journal.pone.0178318

**Published:** 2017-06-02

**Authors:** Caroline L. Poli, Autumn-Lynn Harrison, Adriana Vallarino, Patrick D. Gerard, Patrick G. R. Jodice

**Affiliations:** 1 Department of Forestry and Environmental Conservation, and South Carolina Cooperative Fish and Wildlife Research Unit, Clemson University, Clemson, South Carolina, United States of America; 2 Institute for Parks, Clemson University, Clemson, South Carolina, United States of America; 3 Centro de Investigación y de Estudios Avanzados Unidad Mérida, Mérida, México; 4 Department of Mathematical Sciences, Clemson University, Clemson, South Carolina, United States of America; 5 U.S. Geological Survey, South Carolina Cooperative Fish and Wildlife Research Unit, Clemson University, Clemson, South Carolina, United States of America; Phillip Island Nature Parks, AUSTRALIA

## Abstract

During breeding, foraging marine birds are under biological, geographic, and temporal constraints. These contraints require foraging birds to efficiently process environmental cues derived from physical habitat features that occur at nested spatial scales. Mesoscale oceanography in particular may change rapidly within and between breeding seasons, and findings from well-studied systems that relate oceanography to seabird foraging may transfer poorly to regions with substantially different oceanographic conditions. Our objective was to examine foraging behavior of a pan-tropical seabird, the Masked Booby (*Sula dactylatra*), in the understudied Caribbean province, a moderately productive region driven by highly dynamic currents and fronts. We tracked 135 individuals with GPS units during May 2013, November 2013, and December 2014 at a regionally important breeding colony in the southern Gulf of Mexico. We measured foraging behavior using characteristics of foraging trips and used area restricted search as a proxy for foraging events. Among individual attributes, nest stage contributed to differences in foraging behavior whereas sex did not. Birds searched for prey at nested hierarchical scales ranging from 200 m—35 km. Large-scale coastal and shelf-slope fronts shifted position between sampling periods and overlapped geographically with overall foraging locations. At small scales (at the prey patch level), the specific relationship between environmental variables and foraging behavior was highly variable among individuals but general patterns emerged. Sea surface height anomaly and velocity of water were the strongest predictors of area restricted search behavior in random forest models, a finding that is consistent with the characterization of the Gulf of Mexico as an energetic system strongly influenced by currents and eddies. Our data may be combined with tracking efforts in the Caribbean province and across tropical regions to advance understanding of seabird sensing of the environment and serve as a baseline for anthropogenic based threats such as development, pollution, and commercial fisheries.

## Introduction

One of the primary questions addressed by ecological studies of marine birds is the extent to which foraging locations at sea can be predicted by fine-scale, mesoscale, or large-scale oceanographic features [1–3]. During the breeding season, when individuals are central-place foragers [4], the area accessible for foraging is restricted by the time an animal can be absent from the nest, which ultimately is constrained by lengths of incubation shifts or optimal scheduling of meal deliveries to chicks [4–6]. Most seabirds exhibit bi-parental care; incubation duties are traded off and provisioning is supported by both parents [7]. Provisioning schedules can vary within and among breeding seasons [8, 9] and can be affected by sex-based differences in behavior [8, 10], individual condition [11], chick age [10], or chick condition [12].

The physical environment may influence foraging activities, and while breeding, parent seabirds must be able to efficiently assess habitat patches for prey profitability or risk sub-optimal patterns of attendance or sub-optimal provisioning rates [4, 13]. Marine environments (and hence the underlying fine-scale, mesoscale, and large-scale oceanographic features that characterize these environments) can be highly dynamic in space and time [14, 15]. Given these tendencies, seabirds cannot rely solely on site fidelity to locate prey efficiently. They must be able to sense and process environmental information, often at multiple scales, to search for and potentially locate prey while under a time constraint.

Environmental attributes shown to be associated with prey seeking behavior by seabirds include mesoscale features that characterize Large Marine Ecosystems [16], including currents, eddies, upwelling zones, or fronts [14, 17]. Seabirds, particularly those that appear to possess near-ultraviolet vision e.g. Masked Boobies (*Sula dactylatra*) [18], may be able to detect currents, fronts, and fish using visual cues [19]. A large proportion of investigation on prey-searching and the environment has been conducted in productive temperate systems such as the North Atlantic [17, 20, 21] and Benguela Current [22, 23], or within large ocean gyres such as those in the tropical Pacific [24–26] and Indian oceans [27, 28]. It is not known how well findings from well-studied systems transfer to other understudied systems that may be characterized by substantially different oceanographic conditions.

**Fig 1:**
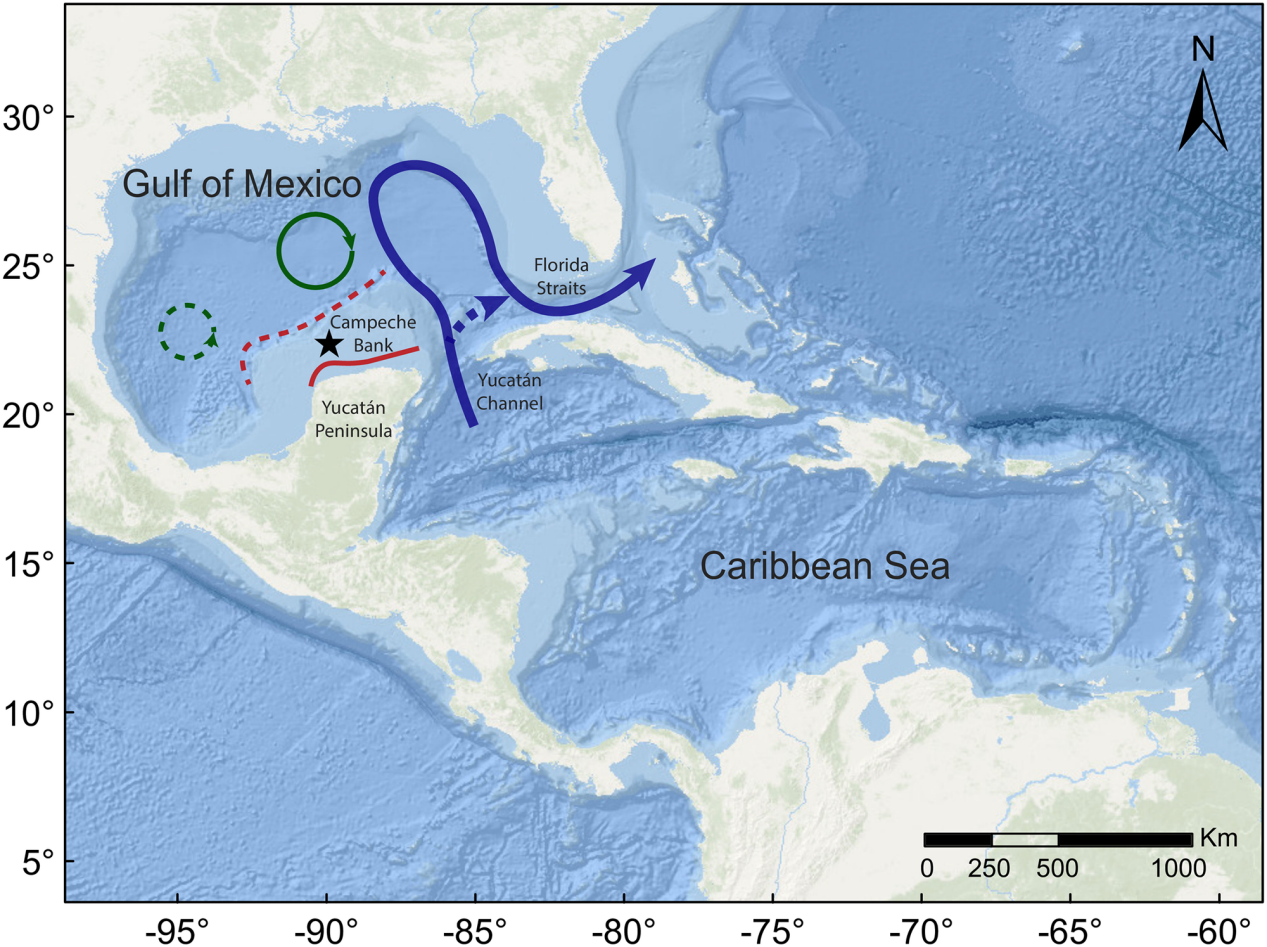
Study location, Isla Muertos (black star) in relation to prominent bathymetric and oceanographic features of the region: Loop Current (blue solid line) and one flow path directly from the Yucatan Channel to the Florida Straits (blue dotted line); anticyclonic (green solid line) and cyclonic (green dotted line) eddies; Shelf-Slope Front (red dotted line); Coastal Front (red solid line). Base map courtesy of ESRI (http://doc.arcgis.com/en/living-atlas/item/?itemId=5ae9e138a17842688b0b79283a4353f6).

Few seabird foraging studies occur in tropical ecosystems that are moderately productive and highly dynamic, including the Caribbean province, a system generally regarded as having an understudied marine avifauna [29, 30]. The two basins of the Caribbean Sea and the Gulf of Mexico (GoM) are considered to be one province due to the extent to which their circulation is integrated [16]. The Yucatan Current, originating in the Caribbean Sea, passes through the Yucatan Channel and then along the Campeche Bank of the southern Gulf of Mexico (Fig 1). Here, it forms the Loop Current, important as the origin of mesoscale features in the GoM, basin circulation, and biogeochemistry [31]. When the Loop Current flows directly from the Yucatan Channel to the Florida Straits, portions of the current separate and form both anticyclonic and cyclonic eddies which circulate throughout the basin [32]. The location and strength of the Loop Current within the GoM can vary within and among years, as can the location and duration of cyclonic and anticyclonic eddies and their associated fronts [31], which often represent productive upwelling zones [33]. Further, the Yucatan Current, Loop Current, and eddies converge and interact with bathymetry to produce a network of thermal fronts, including the Campeche Bank Shelf-Slope and Campeche Bank Coastal fronts, that occur in a pattern that is somewhat seasonal [34]. Seabirds respond positively to frontal zones in other systems in the western north Atlantic [17, 35], but the extent to which birds may respond to mesoscale features within the GoM such as the Loop Current is unclear [29].

Our goal was to explore breeding seabird-foraging dynamics in the understudied tropical western North Atlantic, noting its unique combination of circulation patterns, mesoscale oceanographic features, and bathymetry. We selected the Masked Booby, a pan-tropical seabird that breeds throughout the Caribbean Province and that has been well-studied in other oceanographic systems. Masked Boobies capture prey (primarily flying fish, family Exocoetidae) at or near the surface by plunge-diving [36]. Foraging strategies of Masked Boobies during the breeding season have been investigated at colonies in the western Indian [28], southwest Pacific [9], central Pacific [37], and eastern Pacific [38] ocean basins. Substantial inter-colony differences in foraging ranges, habitat relationships, and individual patterns have subsequently been attributed to inherent differences in the marine environments which range from extremely oligotrophic [28], to oligotrophic [9, 37], to highly productive [38]. Oceanographic factors that are linked to foraging include high net primary productivity (eastern Pacific; [38]), low wind speed (central Pacific; [37]), warm deep water (Indian Ocean; [28]), and a relationship with chlorophyll-a and sea surface temperature that shifts over the course of three breeding phases (western Pacific; [9]). Physical processes may also play a role in structuring foraging ecology of Masked Boobies globally, however, prior studies do not explicitly examine mesoscale currents, eddies, and fronts as determinants of foraging.

Our specific objectives were to 1) Quantify the relative influence of biological and environmental factors in determining foraging behavior, and 2) Determine which oceanographic features predicted prey-searching behavior. We hypothesized that biological factors would influence foraging behavior little compared to environmental factors, and that in the rapidly changing GoM, Masked Booby foraging behavior would be strongly linked to dynamic oceanographic features including eddies and fronts, that tend to concentrate prey. From an ecological perspective, our data will fill a knowledge gap to better understand the relationship between seabird foraging activity and mesoscale oceanographic features in the GoM, allowing comparisons to other systems. From a conservation perspective, our data will provide baseline information on foraging behavior in a system heavily impacted by fishing and offshore oil and gas activities including > 4000 offshore structures [39] and two substantial spill events, each of which affected tropical seabirds [30, 40].

## Methods

### Study area

Data were collected 17–29 May 2013, 1–15 November 2013, and 4–15 December 2014 at the largest breeding colony for Masked Boobies in the north Atlantic, Isla Muertos, Arrecife Alacranes National Park, Mexico (22.4° N 89.7° W). Isla Muertos is located on the Campeche Bank, Mexico, a shelf extension of the Yucatán Peninsula with relatively shallow water, platform reefs, and a steep shelf-slope in the north [41]. The island is a small (0.5 km^2^) coral sand platform reef characterized by exposed bare ground surrounding a core of low-lying succulent vegetation, grasses, and shrubs [42]. Isla Muertos hosts 2500–3000 breeding pairs of Masked Boobies (A. Vallarino, unpublished data). Masked Boobies at this colony breed year-round with peak nesting activity in May-June and November-December (A. Vallarino, unpublished data). Magnificent Frigatebirds (*Fregata magnificens*), Sooty Terns (*Sterna fuscata*), Red-footed Boobies (*Sula sula*), and Laughing Gulls (*Leucophaeus atricilla*) also breed here.

### Data collection

We deployed archival GPS loggers (Mobile Action I-gotU g120) on Masked Boobies during breeding. Tags were removed from the commercial casing and repackaged in heat-shrink tubing to reduce size and weight. We adjusted the frequency with which tags recorded locations multiple times during the study (one location every 10, 33, 50, or 100 s), to optimize battery life. We captured birds during morning or evening from opportunistically selected nests with a handheld net, and attached each tag to the underside of three central retrices with 4–5 strips of Tesa tape. The GPS tag and tape weighed 20.6 grams, corresponding to 1.8% of the mass of the lightest adult measured. During initial capture, adults were sexed by vocalization [43] and marked on the chest and head with non-toxic paint to facilitate later detection. Birds were released at the nest within 15 minutes and quickly resumed attendance. We recaptured GPS-tagged individuals to recover the GPS unit 37–237 hours after deployment which generally resulted in ≥ 1 foraging trip per bird. Each individual was captured/recaptured only once during the study. We counted the number of nests with eggs or chicks and multiplied by two to obtain a count of breeding individuals for each sampling period; we estimated the number of roosting birds located outside of the nesting area and added it to the count of breeding birds to obtain a measure of colony size. We cannot be certain that roosting birds were not associated with a nest, thus our population estimate is potentally an overestimate. The study was approved by the Clemson University Institutional Animal Use and Care Committee (Permit no. 2012009) and permission to work at Isla Muertos was granted by the Comisión Nacional de Áreas Naturales Protegidas (CONANP).

### Data processing

Upon recapture, we removed GPS tags and downloaded tag data. We used the R package *adehabitatLT* [44] to process and analyze foraging trips. Data were grouped into foraging trips beginning with the location immediately prior to colony departure and ending with the location immediately following colony return. Locations that represented unrealistic flight speeds (> 90 km/h; < 20 locations) were removed from further analysis. Data were converted into UTMs and linearly interpolated at 100-meter intervals to meet the requirements of subsequent analyses (delineation of area restricted search; ARS) [45], and to standardize sampling among tags with different data collection frequencies. Multiple foraging trips were collected per individual deployment (range 1–9 trips per bird, mean 2.2). For each trip, we calculated trip duration, total distance traveled, and maximum distance from colony.

Sixty seven percent of trips lasted one day, however, birds spent one night on the water in 30% of trips (2-day trip) and two consecutive nights on the water in 3% of trips (3-day trip). Foraging behavior of Masked Boobies may change from day to day for several possible reasons: 1) the GoM is a dynamic system and the location of prey and oceanographic features associated with prey may change overnight [31, 46, 47], 2) birds that spend the night on the water may drift away from known areas, fishing boats, and conspecifics that could act as cues for prey, and 3) the biological function (i.e. adult maintenance, chick provisioning) of each day of a multi-day foraging trip may differ as prey that are ingested on day one may be digested during the night and over subsequent days [48, 49]. We therefore assessed prey-searching behavior and oceanographic attributes that corresponded to foraging separately for each day of each multi-day trip. Locations collected overnight where birds appeared to be resting on the water for extended periods of time were removed from further analysis of foraging behavior [1, 50], and multi-day foraging trips were then subdivided into bird-days at sea.

### Defining area restricted search locations

**Fig 2:**
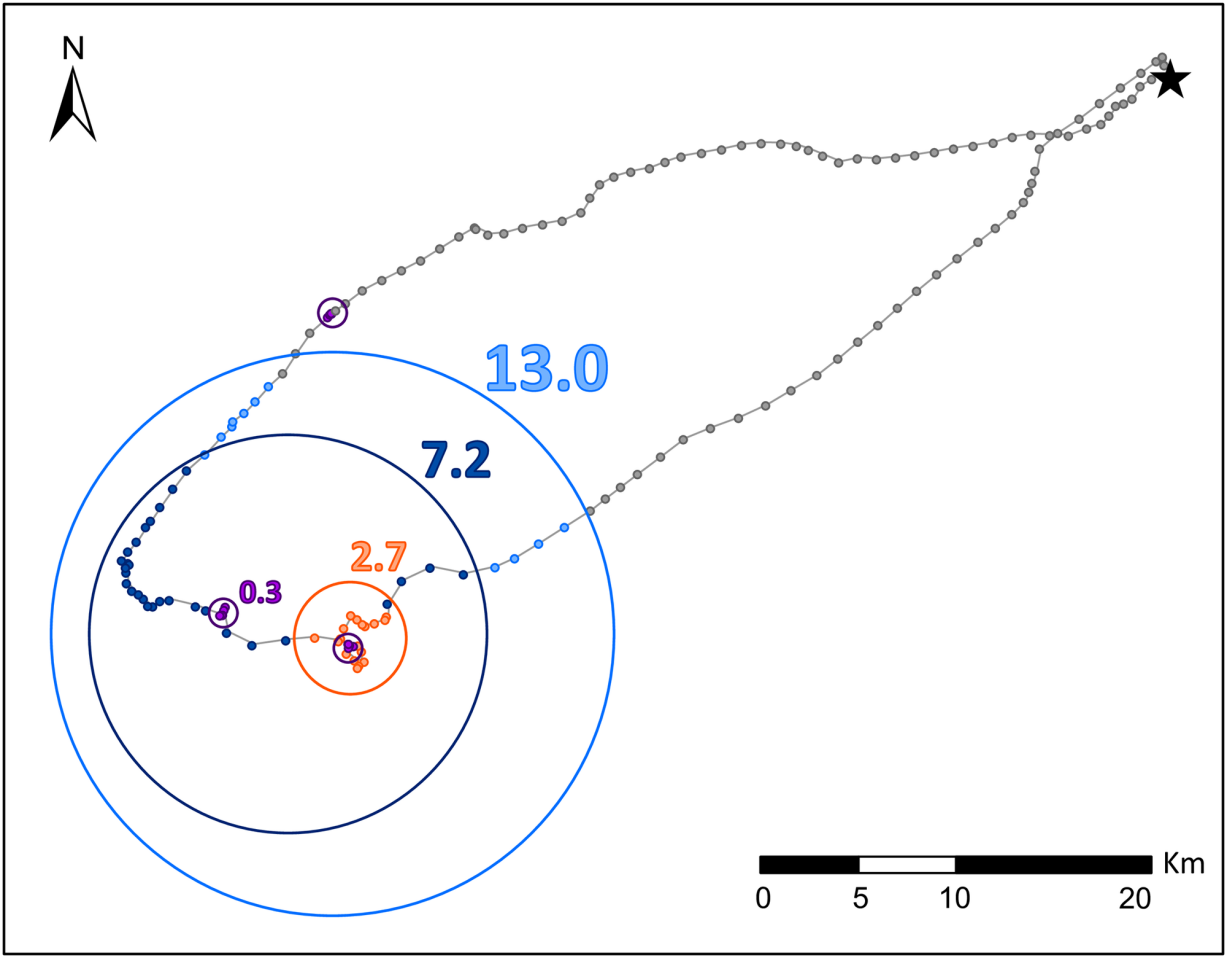
An example of multiple nested scales of area restricted search (ARS) behavior during a single foraging trip. The locations which correspond to foraging at each scale (delineated by colors) are nested from largest to smallest. Points indicate GPS locations, circles and numbers indicate search radii in km.

We took an ARS approach to identify daily locations and scales of foraging effort. Previous studies have shown areas of restricted search to be an indicator of foraging behavior in Masked Boobies because tortuous movements often correspond to areas of prey capture [45, 50]. We took a first passage time (FPT) approach [45] to identify ARS zones during each day’s movement path for each individual. At each location along a movement path, FPT measures the time required for an individual to traverse along the track both forward and backward across a circle with a fixed radius. For each bird-day we repeated the FPT calculation using circles of radii ranging from 10 m (the approximate accuracy of GPS locations) to 35000 m (half the average distance from the colony for all recorded locations), at 10 m increments. The maxima in variance (log(FPT)) were used to identify prominent scales of ARS. In some cases, FPT analysis identified multiple ARS radii within a single day (for example, see Fig 2). We summarized ARS behavior by calculating the number of nested ARS scales and the size of smallest and largest ARS for each bird-day, and averaged across bird-days for each trip. For subsequent fine-scale analysis of foraging behavior and oceanographic habitat use, we selected only the smallest scale of ARS for each bird-day (hereafter, small-scale ARS) [51]. For fine-scale analysis we also eliminated ARS scales > 8 km that likely corresponded to mesoscale search behavior rather than targeted prey-capture.

To designate locations as ARS or non-ARS for each identified scale we used the penalized contrast method of Lavielle [52] which groups locations that have high FPT values and are adjacent to each other along the track. The number of possible segments that could be formed from a track (k-value) was set to a minimum of two and a maximum of 20 following Lavielle [52]. We calculated the number of small-scale ARS zones, duration of small-scale ARS, distance traveled in small-scale ARS, mean distance from colony to ARS, and mean angle from colony to ARS.

### Environmental data

Environmental variables were extracted at the smallest spatial scale at which they were available for each recorded GPS location and linearly interpolated in ArcGIS using Duke University’s Marine Geospatial Ecology Tools [53] unless otherwise specified (Table 1). Daily sea surface temperature (SST; °C) was provided by the Group for High Resolution Sea Surface Temperature (GHRSST). Eight-day and monthly SST were provided by NASA’s Jet Propulsion Laboratory. Chlorophyll-a (Chl-a; mg/m^3^) was derived at two spatio-temporal resolutions from the Moderate Resolution Imaging Spectroradiometer (MODIS) sensor carried onboard NASA’s Aqua satellite. Velocity (m/s) of flowing water was calculated by the National Oceanic and Atmospheric Administration’s (NOAA) Ocean Surface Current Analyses Real Time (OSCAR) project. Bathymetric data (m) were obtained from NOAA’s ETOPO1 Global Relief Model [54]. Oceanic front probability was generated through the NOAA CoastWatch Program by applying an edge detection algorithm to daily SST datasets acquired onboard NASA’s Geostationary Operational Environmental Satellites (GOES) [55]. Frontal data were accessed by querying the NOAA data server, ERDDAP (http://coastwatch.pfel.noaa.gov/erddap). We calculated distance from each GPS location to the nearest front. Daily sea surface height anomaly (SSHA; m above a reference sphere) and mixed layer depth (m; the depth at which the temperature is 0.2°C cooler than the temperature at the surface) were derived from sensors and the Hybrid Coordinate Ocean Model (HYCOM) [56] GoM analysis. Analysis and data collection for SSHA and mixed layer depth were performed by the Navy Coupled Ocean Data Assimilation (NCODA), which assimilates data from satellite sensors collected by the Naval Oceanographic Office (NAVOCEANO) Altimeter Data Fusion Center, satellite and in situ SST measurements, and in situ vertical temperature and salinity profiles from ship-based expendable bathythermographs (XBTs), free-drifting Argo floats, and moored buoys. A blended product for 6-hourly wind speed and direction at the sea surface was provided by the NCODA. The data were accessed by querying ERDDAP and the time of each GPS location was matched with the time at which the wind speed was measured, within 6-hours.

**Table 1:** Physical oceanographic variables and the hypothesized link with foraging behavior of Masked Boobies nesting at Isla Muertos, Mexico, May 2013 and November 2013, and December 2014.

Variable	Temporal resolution	Spatial resolution	Range	Hypothesized link	Citation
Bathymetry (m)	None	0.016°	-3788 - -1	Water circulation and mixing regime influences prey community composition and abundance	Yen *et al*. 2004 [57] Kappes *et al*. 2011 [28]
Chlorophyll-a concentration (mg/m^3^)	8-day	0.05°	0.05–3.4	Recent biogeochemistry linked to prey distribution and abundance	Paiva *et al*. 2010 [58]
Chlorophyll-a concentration	Monthly	0.05°	0.06–2.9	Biogeochemical regime linked to prey distribution and abundance	Perόn *et al*. 2010 [59]
Distance to nearest SST front (km)	14-day	0.05°	0–142.0	Proximity to features that aggregate prey and influence production; visual cue for MABO	Scales *et al*. 2014 [17]
Mixed layer depth (m)	Daily	0.05°	1.0–97.8	Influences activity of subsurface predators and prey and therefore accessibility of prey for MABO	Spear *et al*. 2001 [60] Devney *et al*. 2009 [61]
Sea surface height anomaly (m)	Daily	0.05°	-0.1–0.4	Physical forcing influences prey distribution	Nel *et al*. 2001 [62] Yoda *et al*. 2014 [63]
Sea surface temperature (°C)	Daily	0.01°	23.5–28.2	Immediate thermal conditions linked to prey activity level	Erwin and Congdon 2007 [64] Weeks *et al*. 2013 [65]
Sea surface temperature	8-day	0.05°	24.2–28.0	Recent thermal conditions linked to prey activity level and abundance	Kappes *et al*. 2010 [66]
Sea surface temperature	Monthly	0.05°	24.3–28.2	Thermal regime linked to prey abundance	Renner *et al*. 2013 [67]
Velocity of moving water (m/s)	5-day	0.33°	0.01–0.2	Speed, direction, and magnitude of water movement linked to prey distribution	Schwemmer *et al*. 2009 [68]
Wind speed (m/s)	6-hour	0.25°	2.2–23.0	Influences water mixing linked to prey distribution; affects aerial maneuverability of MABO	Weimerskirch *et al*. 2005 [69] Thorne *et al*. 2016 [70]

### Analysis of at-sea movements and foraging behavior

All statistical analyses were conducted in R 3.0.3 [71]. We used chi-squared tests to determine whether the proportion of single and multi-day trips differed between sampling periods, sexes, and stages. We tested whether foraging behavior metrics differed by sex, nest stage (and their interaction), or sampling period by first using an ordination technique to combine information from multiple foraging behavior metrics and then testing for differences among groups. Foraging behavior metrics included trip descriptors (trip duration, total distance traveled, maximum distance from colony, size of smallest ARS, size of largest ARS; Table 2) and characteristics of small-scale ARS (number of ARS scales, number of ARS zones, duration of ARS, distance traveled in ARS, distance from colony to ARS, and angle from colony to ARS; Table 2). Groups under consideration were sex, nest stage, sampling period, and the interaction between sex and nest stage.

**Table 2:** Summary statistics (mean, sd, range) for foraging behavior of Masked Boobies during incubation and chick-rearing.

Foraging trip descriptors	Incubating Mean ± SD	Chick-rearing Mean ± SD	Incubating Range	Chick-rearing Range
Trip duration (h)	14.6 ± 11.8	10.1 ± 8.5	1.1–51.9	1.0–49.0
Total distance traveled (km)	247.0 ± 128.0	192.3 ± 102.9	36.1–611.9	22.9–557.0
Maximum distance from colony (km)	88.9 ± 40.9	71.6 ± 34.9	15.0–231.5	9.0–211.0
Size of smallest ARS (km)	2.3 ± 3.0	2.2 ± 2.1	0.2–11.7	0.2–15.0
Size of largest ARS (km)	11.1 ± 7.8	9.6 ± 6.7	0.6–31.7	0.3–34.7
**Characteristics of small-scale ARS**				
Number of nested ARS scales	1.9 ± 0.9	2.0 ± 0.9	1.0–6.0	1.0–5.0
Number of ARS zones	2.0 ± 1.2	1.9 ± 1.1	1.0–6.0	1.0–7.0
Duration of ARS (h)	2.0 ± 2.2	2.1 ± 1.6	0.3–8.8	0.2–7.9
Distance traveled in ARS (km)	16.4 ± 11.8	17.3 ± 13.6	1.7–60.6	0.8–68.3
Distance from colony to ARS (km)	87.3 ± 41.6	65.6 ± 32.6	1.8–218.2	2.0–211.1
Angle from colony to ARS (°)	198.6 ± 73.9	182.3 ± 71.8	12.8–341.4	13.7–353.5

We used the R package *vegan* to implement nonmetric multidimensional scaling [NMDS; 71] to first classify observations of Masked Booby foraging behavior. This approach allowed for a high number of dimensions, a variety of data types, and non-linear relationships; it is robust to correlated measurements and suitable for data which do not have an identifiable distribution [72]. We then tested whether the centroid of each of the abovementioned groups differed (e.g. does foraging behavior differ between sampling periods?) using perMANOVA models with 10,000 permutations. We also tested whether variability differed among groups (e.g. is foraging behavior more variable in males compared to females?) using Beta disperser and a permutation test with 10,000 permutations of the model residuals [73]. Beta disperser is an extension of Levene’s test for homogeneity of variance that is useful for multivariate analyses. Post-hoc pairwise comparisons were used to distinguish the relationship between groups.

### Relationship between environment and area restricted search behavior

To estimate the extent to which each environmental variable (Table 1) predicted ARS behavior, we took a random forest (RF) approach [74, 75], which is a bootstrapped classifier algorithm. We conducted a separate RF analysis for each bird-day using R package *randomForest* [76]. For each bird-day we generated a classification tree with a binomial dependent variable (ARS, non ARS) for each location and eleven environmental variables of interest as predictor variables. At each branch of the tree, the environmental variable and corresponding value that resulted in the most accurate partitioning of locations into ARS and non-ARS behavior was selected. The process continued until as many locations as possible were classified, resulting in a final model. The tree was built using a random selection of two-thirds of the locations from each bird-day; the remaining one-third of the locations was used for model validation. To build a RF, the process was iterated 1000 times, resulting in 1000 possible trees for each bird-day. At a given branch, RF analysis randomly selected and considered only three predictors of interest from the pool of eleven (the number of predictors used at each branch was calculated as the square-root of the total number of predictors) [77]. Such sub-setting increases the robustness of RF models to correlation between variables because variables that may be correlated are often separated from one another and thus can be evaluated for performance independently [77]. The Gini index, a measure of the relative contribution of each variable towards formulation of a complete model (i.e. variable importance, measured as the mean decrease in node impurity) [78], was computed across all trees. We extracted the Gini indexes for each variable in each day, then standardized the Gini indexes (for an example of standardized variable importance, see [79]) so that, for each day, the total contribution of all 11 variables added up to 100% (hereafter relative variable importance).

A variable number of bird-days were recorded for each bird (range 1–10, mean 2.6). We therefore used a stratified bootstrap to randomly select one day of data from each trip for each bird, estimate the relative variable importance for each environmental variable in the subsample, and replace the data. The process was iterated 1000 times to generate a population-level mean for each environmental variable. To examine seasonal and interannual differences in ARS behavior we also repeated the bootstrapping process separately for each sampling period. For each bootstrap iteration, we used only one day per bird. We extracted values for partial dependence for each variable and each bird-day and used partial dependence plots with a generalized additive model (GAM) smooth to evaluate the marginal effect of each variable on ARS.

## Results

We recorded 266 foraging trips over 364 tracking days from 135 individuals. Sixty-eight female birds contributed 175 tracking days; 67 male birds contributed 189 tracking days. Twenty-seven females were incubating and 41 were rearing chicks; 24 males were incubating and 43 were rearing chicks. Estimated colony size was 3100 in May 2013, 5000 in November 2013, and 5300 in December 2014.

We recorded at least one complete single-day trip for 75% of birds, at least one 2-day trip for 33% of birds, and at least one 3-day trip for 2% of birds. There was a difference between sampling periods in the number of single-day versus multi-day trips that were recorded (χ^2^ = 10, df = 2, p = 0.007), with a higher proportion of multi-day trips in December 2014 ($\hat{\text{p}}\;=0.33$) compared to May 2013 ($\hat{\text{p}}\;=0.15$) and November 2013 ($\hat{\text{p}}\;=0.18$). Within sampling periods, females and males were equally likely to undertake multi-day trips (χ^2^ < 2.5, df = 1, p > 0.1), and there was also no difference in the proportion of incubating and chick-rearing birds that made multi-day trips (χ^2^ < 2.3, df = 1, p > 0.1).

**Fig 3:**
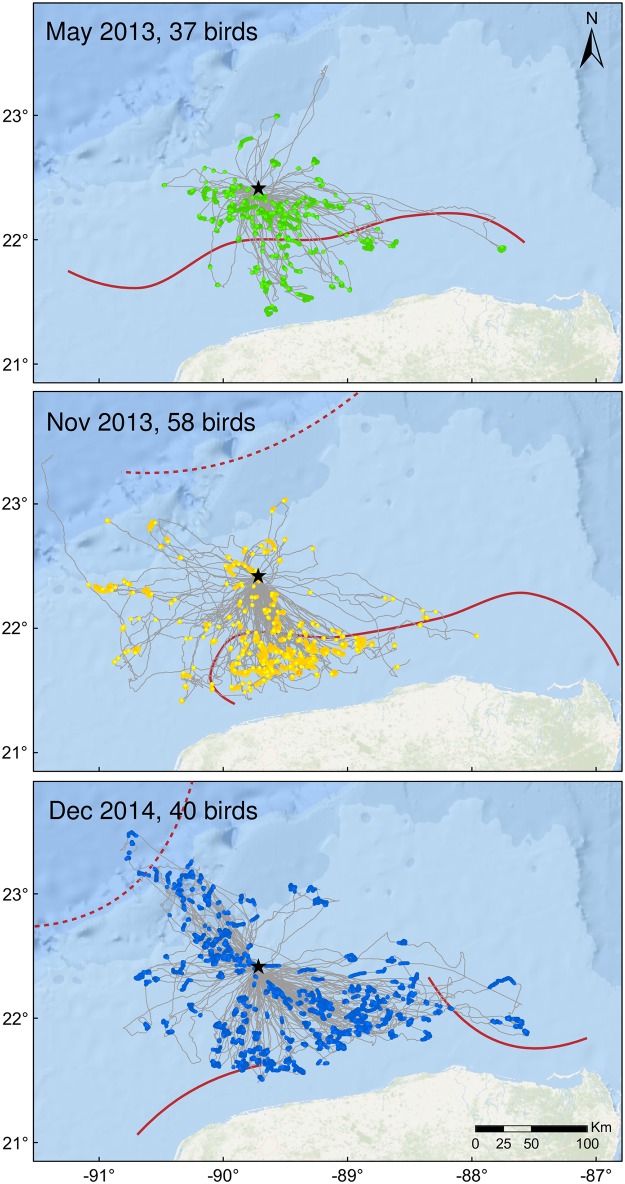
Foraging trips (light gray lines; n = 266 foraging trips over 364 tracking days) and locations of small-scale area restricted search behavior (colored points) identified from first passage time analysis. Foraging trips were recorded from nesting Masked Boobies tracked from Isla Muertos (black star) in May 2013 (19 female, 18 male; 0 incubating, 37 chick-rearing), November 2013 (31 female, 27 male; 41 incubating, 17 chick-rearing), and December 2014 (18 female, 22 male; 10 incubating, 30 chick-rearing). Generalized dominant mesoscale fronts of each sampling period are shown, including the Campeche Bank Shelf-Slope Front (red dotted line) and the Campeche Bank Coastal Front (red solid line).

Foraging locations were located south (angle from colony ARS 157.5°-202.5°) and southeast (202.5°-247.5°) of the colony towards the Yucatán peninsula in all three seasons (26.2% and 27.2% of trips to the south and southeast respectively; Fig 3). In December 2014, birds also traveled northwest (292.5°-337.5°) to the edge of the Campeche Bank shelf in 20.2% of trips (Fig 3). Large-scale oceanography varied considerably between the 3 sampling periods. In May 2013 the Campeche Bank Coastal Front was strong while the offshore Campeche Bank Shelf-Slope Front was absent. In November 2013 both fronts were present but weak. In early December 2014, both fronts were strong but the location of frontal zones changed daily and fronts diminished in extent by mid-month (Fig 3). All three sampling periods were characterized by strong winds from the east-northeast, but in November 2013 thunderstorms occurred frequently and lasted 1–24 hours each.

**Fig 4:**
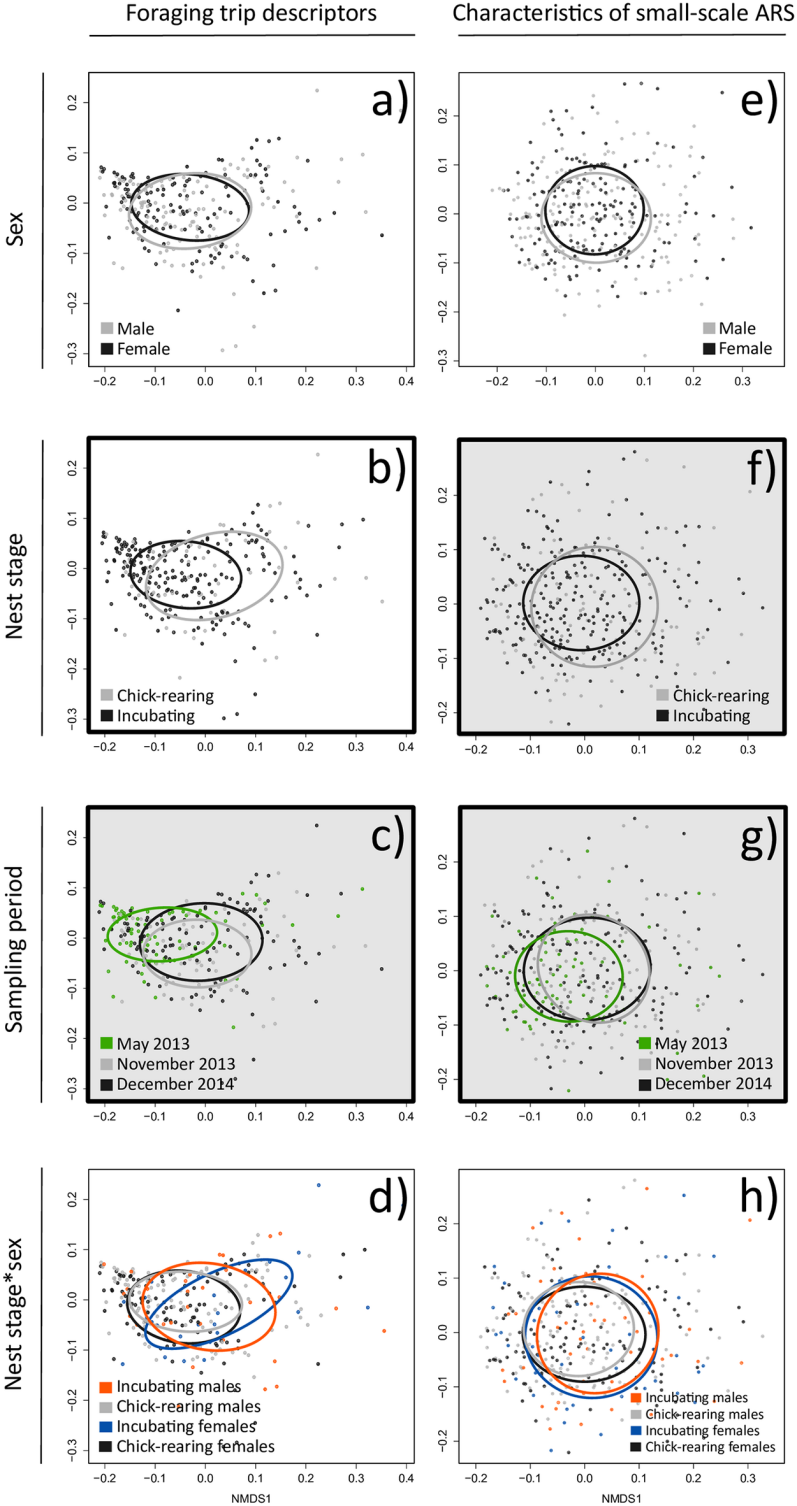
Non-metric multidimensional scaling of foraging trip descriptors (left column a-d) and characteristics of small-scale ARS (right column e-h) by sex (a and e), nest stage (b and f), sampling period (c and g), and sex*nest stage interaction (d and h). Shading represents a significant effect (p < 0.05) as determined by perMANOVA, which tests whether the group centroids differ in multivariate space (e.g. different foraging behavior between sampling periods). A thick black border indicates a significant effect of origin using Betadisperser followed by ANOVA, which tests whether the dispersion of a group from its median is different from the dispersion of other groups (e.g. different relative redundancy in foraging behavior between sampling periods).

Stress values for PerMANOVA of foraging trip descriptors and characteristics of small-scale ARS in relation to sex, nest stage, sampling period, and sex*nest stage were 0.12 (non-arbitrary arrangement of data for each cloud) and 0.24 (somewhat arbitrary arrangement of data for each cloud) respectively [80]. Foraging trip descriptors and small-scale ARS behavior differed between sampling periods (Fig 4c and 4g, Table 3) and incubating and chick-rearing birds also behaved differently in small-scale ARS (Fig 4f). No other significant differences were detected among foraging trip descriptors or small-scale ARS and sampling period and sampling period, nest stage, sex, or sex*nest stage (F ≤ 2.0, p ≥ 0.11 for all; Table 3).

**Table 3:** Results of perMANOVA and permutation tests of Beta dispersion models for five foraging trip descriptors and six characteristics of small-scale ARS that were analyzed in relation to Masked Booby sex, stage, sampling period, and the interaction between sex and nest stage. Significant p-values are in bold; alpha = 0.05.

Foraging trip descriptors	perMANOVA		Beta dispersion	
	Pseudo-F	p-value	F	p-value
Sex	2.00	0.11	0.003	0.96
Nest stage	1.39	0.23	5.39	**0.02**
Sampling period	4.24	**0.002**	3.83	**0.02**
Sex*Nest stage	0.53	0.63	1.76	0.16
**Characteristics of small-scale ARS**				
Sex	1.83	0.15	2.06	0.15
Nest stage	4.93	**0.008**	6.28	**0.01**
Sampling period	7.24	**< 0.001**	6.65	**0.001**
Sex*Nest stage	2.03	0.12	2.34	0.07

We also estimated the Beta dispersion of foraging trip descriptors and characteristics of small-scale ARS in relation to sex, nest stage, sampling period, and sex*nest stage. Foraging trip descriptors and small-scale ARS behavior during chick-rearing was significantly less variable than during incubation (Fig 4b and 4f), however, the relationship with nest stage did not differ between sexes (Fig 4d and 4h). Additionally, foraging trip descriptors and small-scale ARS behavior was more variable in November 2013 and December 2014 compared to May 2013 (Fig 4c and 4g). No other significant differences in Beta dispersion were detected among foraging trip descriptors or small-scale ARS and sampling period, reproductive stage, sex, or sex*nest stage (F ≤ 3.0, p ≥ 0.07 for all; Table 3).

### At-sea movements and foraging behavior

FPT analysis identified at least one ARS in 98% of tracking days. ARS zones ranged in size from 0.2–34.7 km and ARS occurred at multiple nested scales in 64% of bird-days (for example, Fig 2). In 2% of bird-days (n = 8), only mesoscale ARS > 8 km were identified suggesting that birds on these days did not concentrate search effort at any particular small scale; these trips were not associated with a specific sampling period, breeding stage or sex.

### Oceanographic habitat use

When analyzed in combination, the 11 environmental variables we assessed predicted ARS behavior accurately. Validation of the random forest models indicated that the percentage of locations correctly classified for each model from the reserved data was high (99.3 ± 0.8%). Sensitivity (the percentage of ARS locations correctly classified) and specificity (the percentage of non-ARS locations correctly classified) of individual models was also high (sensitivity: 94.7 ± 8.6%; specificity: 99.6 ± 0.5%). However, when considered independently, no single environmental variable predicted ARS behavior consistently, and the relative variable importance for each variable in each day ranged from 0–49.6%.

**Fig 5:**
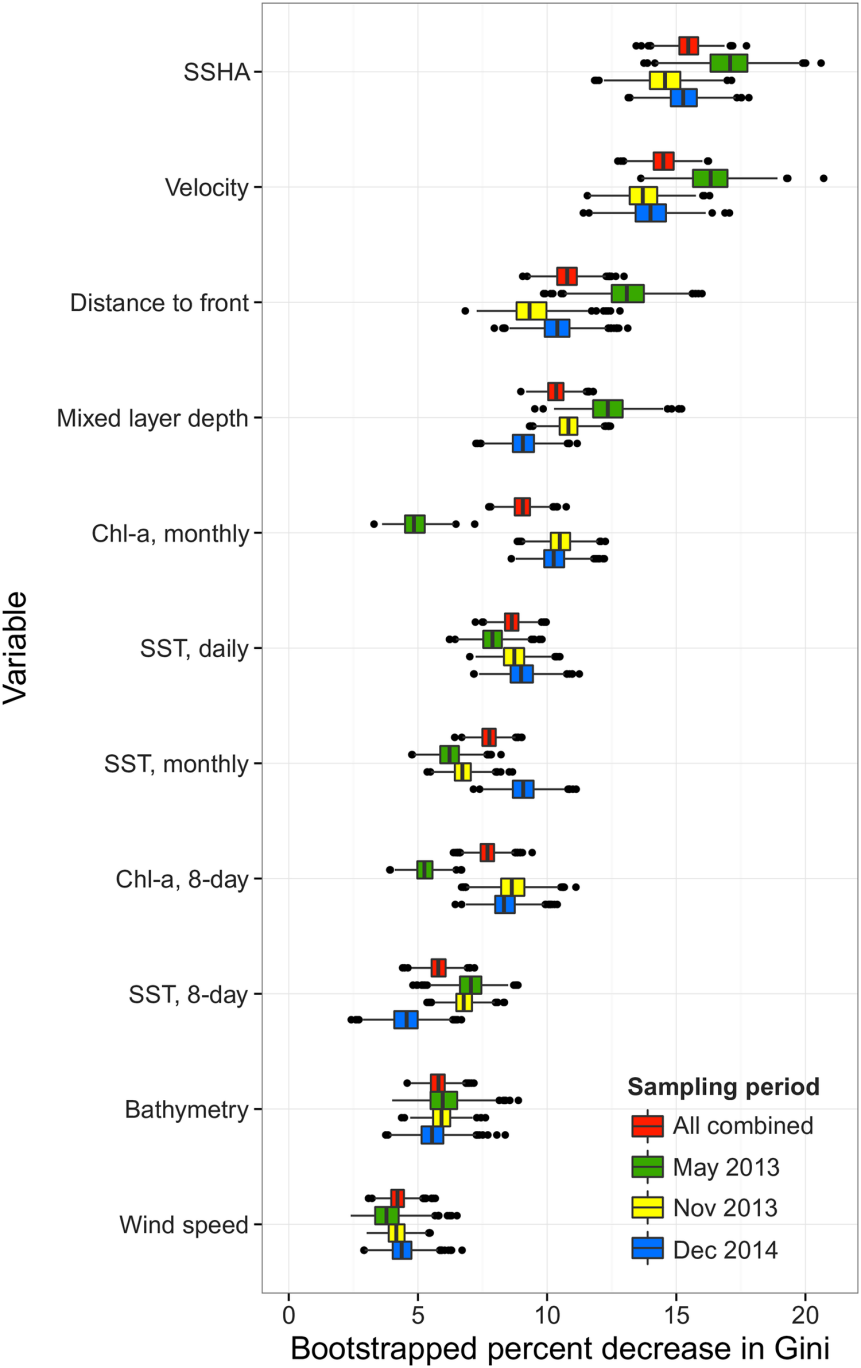
The relative importance of environmental variables as predictors of area restricted search of Masked Boobies nesting at Isla Muertos, Mexico across all sampling periods (red), in May 2013 (green), November 2013 (yellow), and December 2014 (blue). Th N = 135 birds. Each boxplot represents the relative variable importance for a given variable, bootstrapped 1000 times to account for multiple days of foraging contributed by each bird during each trip; central line of each boxplot = mean of means.

**Fig 6:**
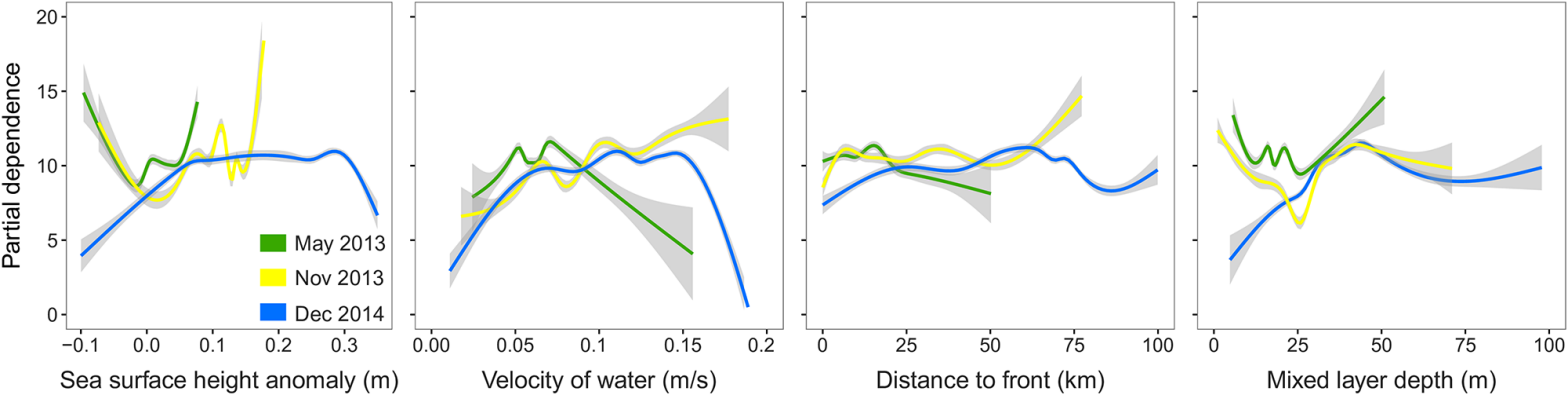
Partial dependence of four variables that were identified as the strongest predictors of area restricted search in random forest models. Lines correspond to GAM smooths of partial dependence of each day during each sampling period: May 2013 (green), November 2013 (yellow), and December 2014 (blue), and shading corresponds to 95% confidence intervals. N = 364 bird-days.

**Fig 7:**
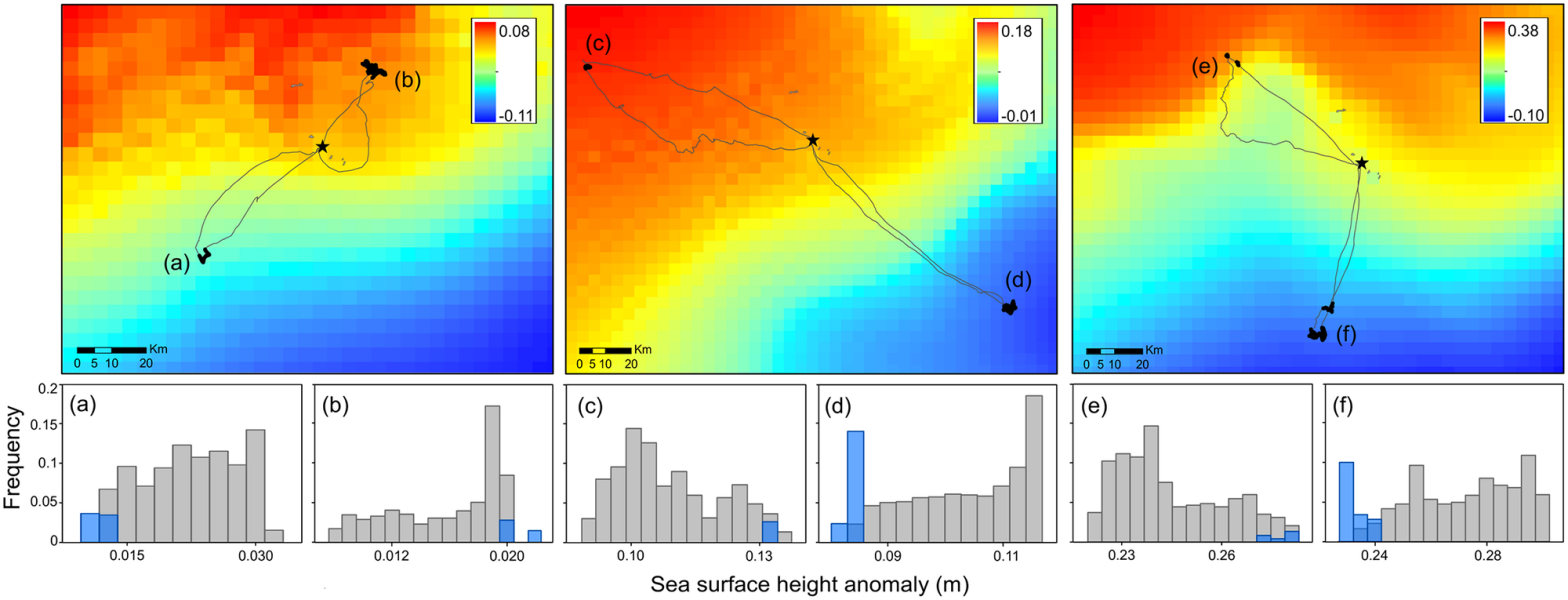
Foraging trips (solid lines) and ARS locations (black dots) of selected individual Masked Boobies overlaid on sea surface height anomaly (SSHA) May 2013 (a, b), November 2013 (c, d), and December 2014 (e, f). Breeding colony = black star. Frequency histograms of SSHA at GPS locations along each of the mapped foraging trips in May 2013 (a,b), November 2013 (c,d), and December 2014 (e,f). Blue boxes = ARS locations; gray boxes = non-ARS locations. Each trip/plot combination corresponds to a unique individual.

Across all bird-days, SSHA and velocity were the strongest predictors of small-scale ARS, contributing 15.6 ± 1.2% and 14.6 ± 1.3% to relative variable importance, respectively (mean of bootstrapped means ± SD; Fig 5). SSHA and velocity were also the most consistent predictors of ARS in the greatest number of individual models: the relative variable importance was higher than the 75^th^ percentile (12.8%) in 58.6% (SSHA) and 53.7% (velocity) of models. Both variables had the highest Gini indexes across sampling periods (Fig 5). The relationship between small-scale ARS and SSHA varied between sampling periods (Fig 6). Low SSHA values < 0 m predicted foraging in May 2013 and November 2013, but middle SSHA values ranging from 0–0.3 m predicted foraging in December 2014. Middle SSHA values of 0.09 m and 0.15 m also predicted foraging in May 2013 and November 2014, respectively. At an individual level, however, small-scale ARS was associated with both low SSHA and high SSHA across sampling periods (for example, Fig 7). Water with a velocity of 0.05–0.1 m/s predicted ARS during all sampling periods (Fig 6).

Distance to front and mixed layer depth also explained ARS within and between sampling periods: relative variable importance was higher than the 75^th^ percentile in 29.4% and 27.4% of models for distance to front and mixed layer depth respectively. At the individual level, foraging occurred at distances to fronts ranging widely from 0–100 km, and small-scale ARS locations occurred within 1 km of ephemeral fronts in 22% of days. In all three sampling periods, foraging occurred where the mixed layer was 35–50 m deep (Fig 6); in May 2013 and November 2013, however, shallow mixed layer depth < 15 m was a better predictor of small-scale ARS than in December 2014.

In 95% of bird-days, two environmental variables had Gini indexes that were higher than the 75^th^ percentile and were identified as important predictors of ARS; in 63% of bird-days, three environmental variables had Gini indexes that were higher than the 75^th^ percentile and were identified as important predictors of ARS. Two-variable combinations that appeared frequently were SSHA + velocity (31.7%), SSHA + mixed layer depth (14.9%), velocity + distance to front (14.3%), and velocity + mixed layer depth (12.3%). There was little consistency among trips in the identity of variables appearing in the three-variable models.

## Discussion

### Oceanographic habitat use and the Gulf of Mexico

Our objective was to predict ARS along each foraging trip using a suite of environmental variables. Across birds, the 11 oceanographic features we tested predicted small-scale ARS accurately (sensitivity, specificity, and percentage of locations correctly classified was high), but among birds, the identity and strength of the oceanographic features that predicted ARS were variable (Fig 6). Thus we predicted ARS at the population level but the relationships that lead to that prediction are not consistent within individuals. In this study, direct measures of dynamic oceanographic topography (SSHA and water velocity), were the strongest predictors of ARS (Fig 5). Ocean currents circulate around and within peaks and troughs created by areas of high and low sea surface height, forming small and large-scale features of ocean surface topography including eddies, currents, and gyres. Several possible mechanisms may link SSHA and velocity to Masked Booby foraging behavior (Figs 5 and 6). First, boobies appear to possess color and near-ultraviolet vision [18] suggesting they have the ability to detect the visible edge that forms when water bodies that differ in turbidity and nutrient levels converge [81]. They may also use visual cues such as areas of low turbulence and slow-moving currents (detectable due to the transport of floating macro-algae and debris) to locate prey. Second, SSHA and velocity are measurements of the physical motion of horizontal currents which create habitat that influences the distribution of prey. Fish, and therefore birds, may associate with multiple types of currents, leading to a range of relationships between foraging and oceanography (Figs 6 and 7). For example, in the northern GoM, areas of low SSHA and velocity provide habitat for spawning flying fish, a major prey item for Masked Boobies, by allowing for suspension and transport of fish eggs or larvae within the water column [82]. This is in contrast to the South Atlantic Bight where areas of high SSHA within warm core rings concentrate floating *Sargassum* (a macro-algae) and fish, which attracts seabirds [35, 83]. Finally, at the edges of eddies where the SSHA and velocity gradient is sharp, surface currents may passively collect and transport fish eggs, larvae, and adults [84], attracting birds. The GoM is a topographically dynamic ecosystem dominated by frequent mixing [16], and the link between seabird foraging and SSHA may vary with the stability of a given region and the prey-sensing mechanisms used by a given species.

In many systems, foraging behavior in seabirds is influenced by large-scale features and processes related to SST that tend to concentrate prey, such as the North Pacific Transition Zone [66], the Atlantic Gulf Stream [35], or mesoscale convergence zones in the Indian Ocean [85] at multiple time scales (i.e., daily, annual, decadal) [86]. Neither SST nor frontal features related to SST were of high relative importance in a large proportion of individual models relative to other variables, even when considered at multiple temporal scales (i.e. daily, 8-day, 14-day, monthly). In the GoM, temperature-related features may not be as reliable predictors of prey presence compared to other important oceanographic features. Frequent mixing by the Loop Current and associated eddies may limit the long-term persistence of large-scale SST features and related fronts, leading to an ecosystem that is highly dynamic and resulting in a weak or inconsistent relationship between temperature-associated features, prey fish, and foraging locations. SST is a complex variable which is influenced by topography and integrates factors including solar heating, runoff, and precipitation [16] that may not necessarily be linked to prey. In this study, small-scale ARS locations occurred within 1 km of ephemeral fronts in 22% of days, and the location of seasonal fronts appears to influence the overall direction of trips (Fig 3). However, direct measures of dynamic oceanographic topography predicted ARS 3.2–5.3 times more often than habitat descriptors such as SST (Fig 5). It is also possible the chain of production in the GoM, from nutrient mixing to prey maturation occurs at a time-lag other than those considered in this study. The presence of such a time-lag could lead to a temporal mismatch between SST-related features and foraging locations of Masked Boobies which correspond to prey presence [22, 87].

### How might local conditions have influenced foraging behavior?

Foraging behavior in May 2013 was less variable than in November 2013 and December 2014. In the GoM in November-January, strong westerly winds drive upwelling along the Campeche Bank shelf [88]. The shelf-slope front (Fig 1) forms as a result of winter mixing but changes position and may dissipate entirely in summer, resulting in seasonal patterns that likely influence the distribution and abundance of prey fish near the shelf. In contrast, the coastal front (Fig 1), which forms when topography, tide, and wind generate nearshore upwelling of cold water, was consistently strong during all three sampling periods, although the location and distance from shore varied (Fig 3). Seabirds respond to intra- and inter-seasonal differences in prey availability by changing the location, distance, and duration of foraging trips. For instance, Masked Boobies breeding at Phillip Island, Australia traveled to distant feeding grounds during periods when prey patches near the colony were small [9]. It is possible that on Campeche Bank, the formation of the shelf-slope front resulted in a higher proportion of foraging trips to the northwest in December 2014 while foraging trips to the more consistent coastal front in the south-southeast occurred frequently during all three sampling periods (Fig 3).

High levels of intraspecific competition can cause prey depletion near the colony and lead to larger foraging ranges [89–91]. For example, colony size determined trip duration of Northern Gannets (*Morus bassanus*) breeding at sites throughout the United Kingdom, and the effect of density-dependence on foraging behavior was predictable between colonies within a breeding season and also at a single colony across multiple breeding seasons [90, 91]. Therefore, it appears that the number of individuals present at a given colony can determine the foraging radius, apparently even between breeding seasons. The number of birds present at Isla Muertos varied between sampling periods with fewer individuals in May 2013 compared to November 2013 and December 2014, and it is possible that density-dependence led to differences in foraging behavior during periods with fewer birds present.

Regionally abundant commercial, artisanal, and sport fisheries create an opportunity for birds to recruit to fishing boats where they may scavenge discards (sensu [92, 93]) or capture prey that are flushed from the water by boats (i.e., flying fish) [94]. On Campeche Bank, fishing fleets change gear opportunistically within a single trip and target a broad range of species including red grouper (*Epinephelus morio)*, black and yellowmouth grouper (*Mycteroperca* spp.), red snapper (*Lutjanus campechanus*), banded rudderfish (*Seriola zonata*), and octopus (*Octopus* sp.) [95]. As a result of concurrent fisheries, boats commonly operate throughout the year in water depths that range from 20–100 meters [95, 96], which overlaps considerably with the nearshore foraging locations of Masked Boobies observed in all three sampling periods of this study. Although Masked Boobies in the tropical Pacific do not typically recruit to or scavenge discards from fishing vessels [25, 97], whether birds on Campeche Bank respond to the heterogeneous fisheries in the region is unknown. Finally, intra-annual differences in fisheries activity [95] could lead to changes in the type and number of boats that birds can follow, thereby contributing to seasonal differences in foraging behavior of Masked Boobies.

### Biological factors influence foraging behavior

Foraging behavior during incubation was more variable compared to chick-rearing. Trade-offs associated with meeting the energetic demands of both parent and offspring during chick-rearing often produce a shift in behavioral patterns between incubating and chick-rearing stages such that behavioral patterns are more consistent during chick provisioning [98]. Attendance at the nest, and hence the duration of foraging trips, also can be influenced by predation rates at the nest. In situations where predation at the nest is high, parents may invest more time in nest or chick guarding [99]. At Isla Muertos, predation of masked booby chicks (but not eggs) by breeding Magnificent Frigatebirds is common (C. Poli, personal observation), and unguarded chicks could also be attacked by Masked Boobies from neighboring territories [100]. Nest defense during chick-rearing may therefore be an important component of individual reproductive success and a strong driver of differences in foraging behavior between incubating and chick-rearing birds, leading to foraging behavior that is less flexible during the chick-rearing stage.

Foraging trip descriptors and foraging behavior within trips differed little between sexes. It is possible that sex-based differences in foraging behavior may be suppressed by resource depletion near breeding colonies, resulting in increased foraging ranges for both sexes throughout the breeding period. If behavioral differentiation between sexes does exist, it should occur under conditions where competition between birds is low (i.e. small colony size) and prey are abundant. For example, supplemental feeding of breeding Black-legged Kittiwakes (*Rissa tridactyla*) lead to higher energy expenditure in males but not in females, suggesting that increased prey availability releases birds from energetic constraints and allows the sexes to engage in activities that differ depending on parental roles [101]. Studies of breeding Masked Boobies in the western Indian Ocean, central Pacific, and eastern Pacific found little evidence for differentiation in foraging behavior between sexes but were restricted to small colonies (10–300 pairs) in unproductive environments [28, 37, 102], or extremely large colonies (> 100,000 pairs) in highly productive environments [100]. In the former, low intraspecific competition but limited places to forage results in overlapping use of a small area and hence lack of differentiation between the sexes. In the latter, an abundance of suitable foraging locations may allow for flexibility in behavior between sexes, but high intraspecific competition could result in lack of differentiation.

Alternately, local enhancement in which birds follow conspecifics to prey patches, could result in similar behavior for birds of both sexes. Productivity in the southern GoM is moderate [16], and foraging trips recorded during this study were distributed across multiple locations (Fig 3), however high intraspecific competition and/or high local enhancement may have suppressed differentiation between sexes. Differentiation in the depths utilized by each sex could also occur within foraging patches, as has been shown in the eastern and western Pacific where female Masked Boobies rearing chicks dive deeper and more frequently than males [100, 102], and also deliver significantly more food to the chick [100]. Whether differentiation during foraging at Isla Muertos occurs within foraging patches is unknown.

### Scales of ARS

The nested ARS scales observed within individual foraging trips (Fig 2) are consistent with hierarchical organization of patches within a given environment and multi-scale search behavior of foraging seabirds. For example, Kotliar and Wiens [103] described a framework for understanding the scales at which patches occur within a given landscape in which individual fish are distributed within fine-scale aggregations of prey, which are distributed within coarse-scale patches, which are subsequently nested within large-scale regions. Additionally, Fauchald and Tveraa [51] predicted that foraging seabirds would search for prey at multiple scales, following the structure of nested patches within the environment. For example, large scales of 10–40 km may correspond to the farthest distance at which Masked Boobies can search. Haney et al. [104] provide evidence that such a range would correspond closely to the radius of visibility Masked Boobies can search, as observed in other seabird species at a given altitude. Scales between 1 and 10 km may correspond to search effort within an area of suitable habitat where prey encounter is likely. Small search scales < 1 km may correspond to the approximate size of the moving prey patch. Tremblay *et al*. [105] suggest relatively similar scales of movement based on empirical observations of foraging behavior in Cape Gannets (*Morus capensis*). During foraging, each nested scale may correspond to a different set of sensory cues which are used stepwise with changing search scales to locate prey.

FPT analysis did not identify a small scale of search for all bird-days, suggesting that birds that did not have small search scales either did not forage or adjusted the amount of time spent at each small scale of search evenly and continuously during foraging trips. Conversely, birds that searched at multiple nested scales may have done so concurrently, integrating multiple environmental and sensory cues. Foraging seabirds may, for example, scan the proximate eddy for prey and at the same time search the distant horizon for conspecifics [51, 105]. Large gaps exist in scientific understanding of seabird perception and sensing of the environment and prey, however, recent research on Procellariiformes suggests that burrow-nesting seabirds rely on olfaction to locate prey while ground-nesting seabirds primarily use vision [106].

## Conclusions

This research emphasizes that populations of birds may adjust to dynamic environments by using a wide range of oceanographic features to locate prey at fine scales, and that the relationship between seabird foraging and oceanography may therefore vary between regions. Oceanographic features unique to the GoM were most strongly linked to foraging and prey, whereas among individual attributes, nest stage contributed more to individual differences in foraging behavior than sex. Moving forward, studies that link the observed habitat associations and individual behavioral patterns with vital rates could identify specific factors that regulate the large and regionally important population at Isla Muertos. Ultimately, such information can be applied constructively to guide conservation and management of a number of seabird species and serve as a model for understanding the behavior of top predators region-wide.
